# Staphylococcal Toxic Shock Syndrome Associated With a Cosmetic Rhinoplasty Procedure

**DOI:** 10.7759/cureus.87549

**Published:** 2025-07-08

**Authors:** Nanako Kitagawa, Daichi Umemoto, Hiroaki Nishioka

**Affiliations:** 1 Department of General Internal Medicine, Kobe City Medical Center General Hospital, Kobe, JPN

**Keywords:** cosmetic rhinoplasty, fever, hypotension, skin rash, staphylococcus aureus, toxic shock syndrome

## Abstract

Staphylococcal toxic shock syndrome (TSS) is a rare but life-threatening condition. Here, we present a case of a 25-year-old woman who developed staphylococcal TSS following cosmetic rhinoplasty. Two days before her hospital admission, she had undergone cosmetic rhinoplasty and experienced fever, vomiting, and skin rash following the procedure. On admission, she presented with hypotension. TSS was suspected, and treatment comprising fluid resuscitation, vasopressors, and antibiotics was promptly initiated. On examination, methicillin-susceptible *Staphylococcus​​*​​​​​ *aureus* was identified in nasal swab specimens. She recovered within a few days, and desquamation was observed later. Although rare, TSS should be recognized as a serious complication of cosmetic surgeries, particularly given the significant increase in the number of such procedures worldwide.

## Introduction

Toxic shock syndrome (TSS) is a rare but life-threatening complication that can arise from certain bacterial infections. It is primarily caused by toxins produced by *Staphylococcus aureus*, particularly toxic shock syndrome toxin-1 (TSST-1); however, the condition may also be provoked by toxins produced by group A streptococci [1]. TSS typically presents with a sudden onset of fever, rash, hypotension, and involvement of multiple organs. Dermatological symptoms include diffuse macular erythroderma, followed by skin peeling (desquamation) one to two weeks later. Importantly, TSS can affect anyone, even individuals who are otherwise healthy.

Staphylococcal TSS is generally categorized into two types: menstrual and non-menstrual. Menstrual TSS is often linked to tampon use, although there have been reports of its association with menstrual cups and intrauterine devices [1,2]. Non-menstrual TSS may occur in various clinical situations, including surgical and postpartum wound infections, septorhinoplasty, osteomyelitis, purpura fulminans, and post-influenza complications [2,3]. However, reports on staphylococcal TSS after cosmetic rhinoplasty are rare.

In this report, we present the case of a young woman who developed staphylococcal TSS, which was associated with cosmetic rhinoplasty.

## Case presentation

**Figure 1:**
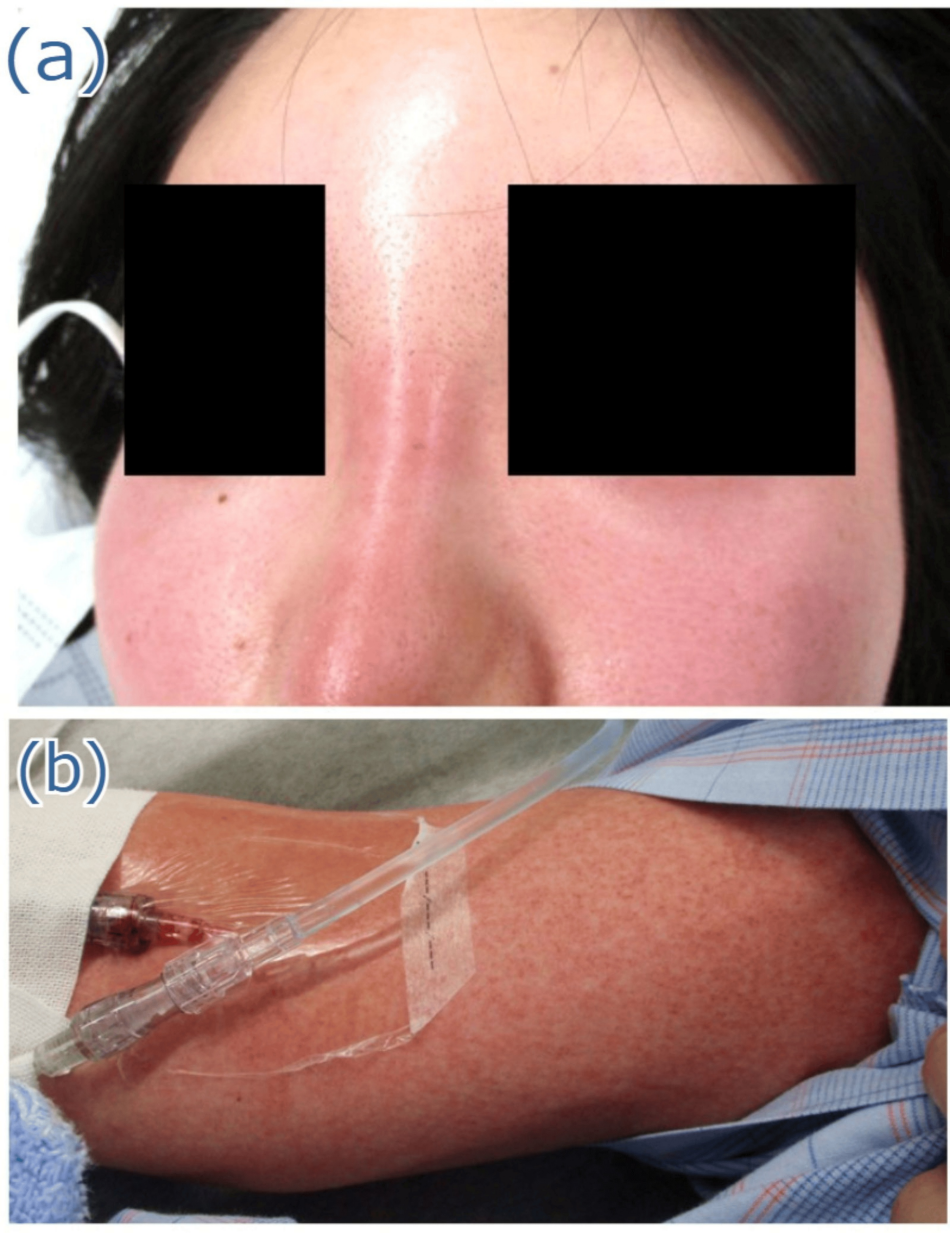
Physical examination findings Redness of the nasal root (a) and diffuse macular erythroderma over the arm (b) are observed.

**Figure 2:**
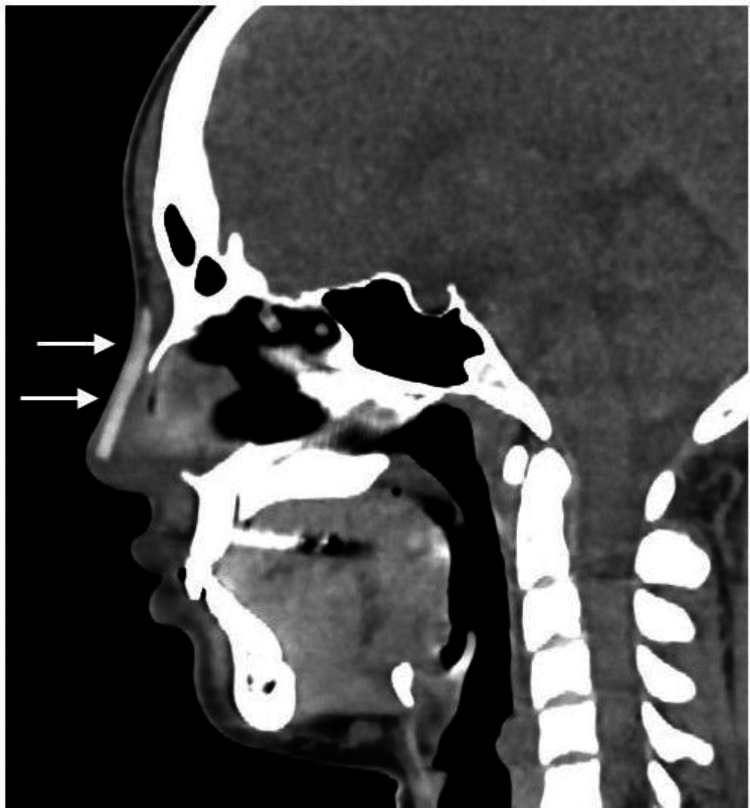
A computed tomography scan of the face A rhinoplasty artifact on the nasal dorsum (white arrows), without surrounding fluid accumulation, is revealed.

A previously healthy 25-year-old woman was admitted to our hospital with a one-day history of fever, vomiting, and generalized skin rash. Two days before admission, she had undergone cosmetic rhinoplasty at another cosmetic surgery clinic. The patient did not take any medication and reported no history of smoking, alcohol consumption, or food or drug allergies. Also, she did not report using menstrual tampons. Physical examination revealed a blood pressure of 69/44 mmHg, pulse rate of 158/min, respiratory rate of 25 breaths/min, and body temperature of 39.3 ℃. The patient was alert and conscious. Notable findings included redness of the nasal root, bilateral conjunctival hyperemia, and diffuse macular erythroderma over the entire body (Figure 1). The heart and breathing sounds were clear, and there were no insect bites. Laboratory findings showed the following results: white blood cell (WBC) count, 13,600/μL (with neutrophils: 92.0%); hemoglobin, 9.8 g/dL; platelet count, 8.6×10^4^/μL; albumin, 3.2 g/dL; aspartate aminotransferase, 38 U/L; alanine aminotransferase, 28 U/L; blood urea nitrogen, 36.8 mg/dL; creatinine, 1.48 mg/dL; C-reactive protein, 28.59 mg/dL (Table 1). Anti-measles IgM antibody test results were negative, and blood and urine cultures did not show bacterial growth. Computed tomography of the face revealed a rhinoplasty artifact on the nasal dorsum, without surrounding fluid accumulation (Figure 2).

**Table 1:** Laboratory findings on admission AST: aspartate aminotransferase, ALT: alanine aminotransferase, BUN: blood urea nitrogen, Cre: creatinine, CRP: C-reactive protein

Blood work	Result	Reference range
White blood cell (/μL)	13,600	3,900-9,800
Neutrophils (%)	92	26-71
Lymphocyte (%)	3.5	19-61
Hemoglobin (g/dL)	9.8	11.1-15.1
Platelet (×10^4^/μL)	8.6	13.0-37.0
Albumin (g/dL)	3.2	3.9-4.9
AST (U/L)	38	8-40
ALT (U/L)	28	8-40
BUN (mg/dL)	36.8	8.0-20.0
Cre (mg/dL)	1.48	0.40-1.80
CRP (mg/dL)	28.59	0.00-0.50

**Figure 3:**
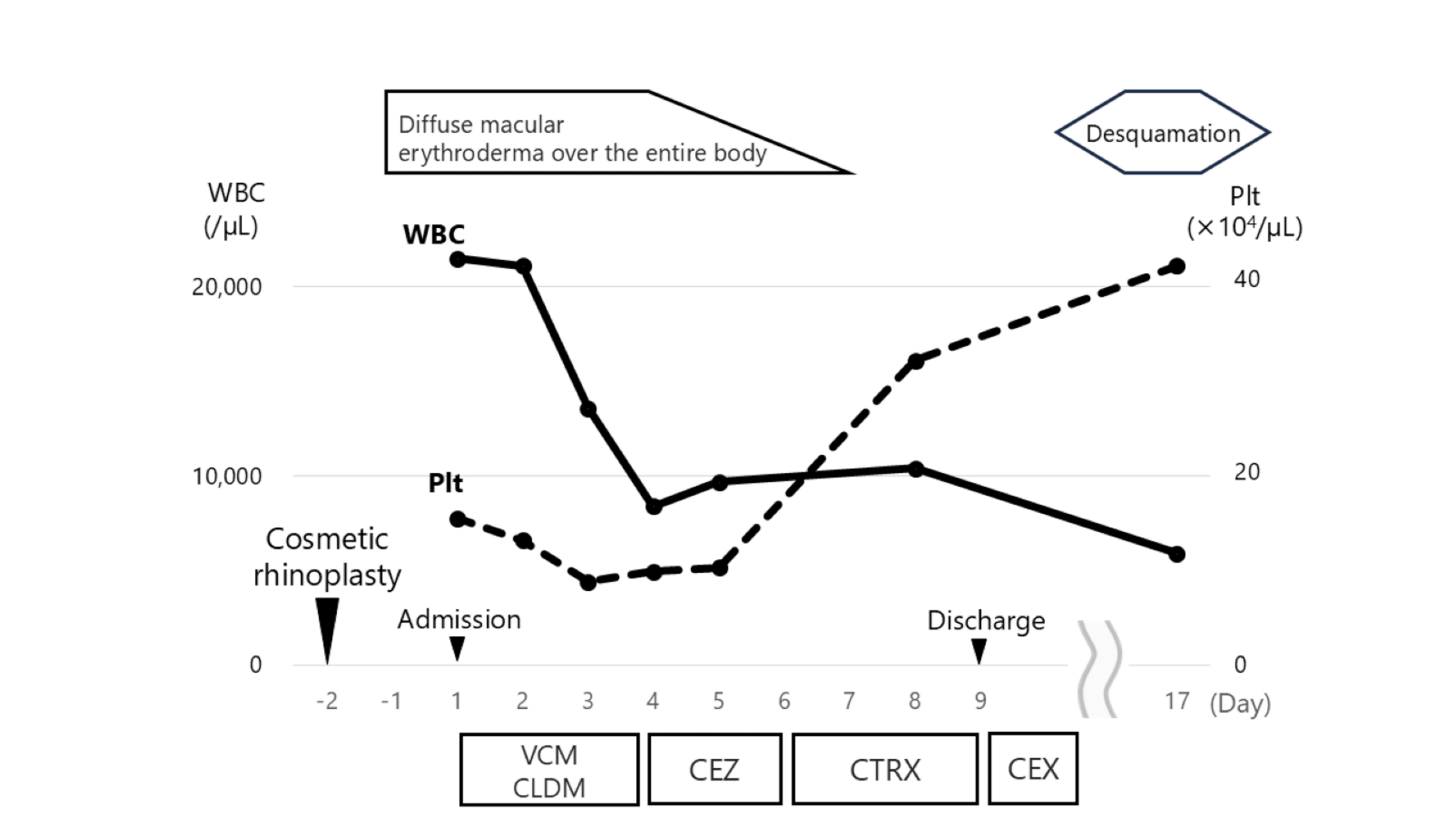
Patient’s clinical course CEX: cefalexin, CLDM: clindamycin, CTRX: ceftriaxone, Plt: platelet, VCM: vancomycin, WBC: white blood cell

TSS was suspected based on clinical findings, including high fever, diffuse rash, hypotension, thrombocytopenia, and vomiting. The patient underwent immediate fluid resuscitation using norepinephrine and vasopressin. Treatment with vancomycin (targeting trough levels of 15-20 µg/mL) and clindamycin (600 mg every 8 h) was initiated. Polymerase chain reaction analysis (FilmArray®; bioMérieux, Tokyo) of nasal swab specimens obtained from the rhinoplasty surgical site tested positive for *S. aureus*, which was identified as methicillin-susceptible *S. aureus*. By day 3, the patient recovered from shock, and the antibiotic regimen was changed to cefazolin (2 g every 8 h) (Table 2). On day 4, the fever resolved, and by day 6, the rash gradually improved. Owing to the increase in eosinophils (WBC count of 9,700/μL with 16.5% eosinophils), likely caused by an allergy to cefazolin, the antibiotic was switched to ceftriaxone (2 g every 24 h). The patient was discharged on day 9. She started taking cefalexin (2 g/day) but discontinued it at her own discretion owing to the appearance of a skin rash. After discontinuation of the medication, the skin rash quickly disappeared, and the patient remained symptom-free and healthy. On day 17, the patient returned to our outpatient department for a follow-up visit, where desquamation of her upper arms was observed; however, there was no redness at the nasal root (Figure 3). Ultimately, the patient was diagnosed with staphylococcal TSS, which is likely related to cosmetic rhinoplasty.

**Table 2:** Antibiogram for MSSA, at our hospital (2024) MSSA: methicillin-susceptible *Staphylococcus aureus*, PCG: penicillin G, ABPC: ampicillin, CEZ: cefazolin, CFX: cefoxitin, CTRX: ceftriaxone, GM: gentamycin, CLDM: clindamycin, LVFX: levofloxacin, VCM: vancomycin, ST: sulfamethoxazole-trimethoprim, LZD: linezolid, DPC: daptomycin

	PCG	ABPC	CEZ	CFX	CTRX	GM	CLDM	LVFX	VCM	ST	LZD	DPC
MSSA	9	9	100	100	100	80	79	83	100	100	100	100

## Discussion

The clinical course of our patient highlighted two important observations: first, TSS may develop after rhinoplasty for cosmetic reasons, and second, TSS can be treated effectively without the need to remove foreign bodies.

TSS is a life-threatening condition that can develop rapidly; therefore, prompt identification of its cause is essential. While tampon use during menstruation is a well-known trigger for TSS, research shows that at least half of the reported cases of staphylococcal TSS are unrelated to menstruation [4], and the mortality rate of non-menstrual TSS can be as high as 4%-22%, which is greater than that of menstrual TSS [1]. Surgery on the nasal area can lead to staphylococcal TSS, and some cases of staphylococcal TSS related to nasal surgery have been reported in the literature (Table 3) [5-16]. However, only one case, documented in French, was associated with cosmetic purposes [11], as seen in our case. Although cosmetic surgery may be less likely to result in staphylococcal TSS, TSS should be recognized as a serious complication of cosmetic rhinoplasty, especially considering the significant increase in cosmetic plastic surgeries globally in recent years, with rhinoplasty procedures alone increasing by 21.6% annually [17].

**Table 3:** Review of staphylococcal TSS related to nasal surgery TSS: toxic shock syndrome, M: male, F: female

Author, year	Age	Sex	Surgical procedure	Detection sites of *S. aureus*	Outcome
Jacobson & Kasworm, 1986 [5]	27	F	Septoplasty	Maxillary sinus	Recovered
	34	M	Septoplasty	(-)	Recovered
	29	F	Septoplasty	Right and left nostril	Recovered
Tobin et al., 1987 [6]	29	F	Herniorrhaphy and septorhinoplasty	Nasal cultures	Recovered
Wilson et al., 1987 [7]	39	F	Antroscopy and turbinectomy	Nasal swab	Recovered
Nahass & Gocke, 1988 [8]	32	M	Nasal polypectomy, septoplasty	Nares and sinus drainage	Recovered
Jones & MacRae, 1990 [9]	50	M	Septoplasty	Nasal cavities	Recovered
Liu et al., 1990 [10]	32	M	Submucous resection of the nasal septum and polypectomy	Throat swab	Recovered
Abifadel et al., 1990 [11], article in French	24	M	Cosmetic rhinoplasty	(+)	Recovered
Schweitzer et al., 1990 [12], article in Dutch	30	M	Septorhinoplasty	Blood culture	Recovered
Abram et al., 1994 [13]	30	M	Functional endonasal sinus surgery, septoplasty	Nasal culture	Recovered
	32	F	Functional endonasal sinus surgery	Nasal culture	Recovered
	14	F	Second-stage endonasal clean-out procedure after functional endonasal sinus surgery	Nasal and throat cultures	Recovered
	25	F	Functional endonasal sinus surgery	Throat cultures	Recovered
	8	M	Second-stage endonasal clean-out procedure after functional endonasal sinus surgery	Nasal cultures	Recovered
Miller & Stankiewicz, 1994 [14]	61	F	Endoscopic bilateral total ethmoidectomy, sphenoidotomy, maxillary antrostomy, and septoplasty	Sinus cultures	Recovered
Graham et al., 1995 [15]	29	M	Nasal septoplasty	Wound cultures	Recovered
Younis & Lazar, 1996 [16]	5	M	Functional endonasal sinus surgery	Direct sinus cultures	Recovered
	7	F	Functional endonasal sinus surgery	Sinus cultures	Recovered
	32	M	Functional endonasal sinus surgery	Blood and sinus cultures	Recovered
Our case	25	F	Cosmetic rhinoplasty	Nasal swab	Recovered

The exotoxin TSST-1, produced by* S. aureus*, is believed to play a significant role in the pathogenesis of staphylococcal TSS. However, the mechanisms underlying postoperative staphylococcal TSS are poorly understood. In this case, the patient had a silicone implant inserted for prosthetic rhinoplasty in the nasal dorsum, where *S. aureus *colonization was likely to occur. Surgical procedures create an oxygen-rich environment in the nasal dorsum that promotes *S. aureus *growth and TSST-1 production. This toxin disrupts the mucosal barrier and stimulates cytokine production, leading to the development of TSS [2]. TSST-1 may also promote *S. aureus* colonization within the nasal dorsum by activating CD8+ T cells [18]. Additionally, surgical procedures compromise the immunological barrier, thereby possibly allowing foreign materials to become substrates for bacterial growth.

The management of TSS includes supportive care, surgical debridement (if warranted), removal of infected foreign bodies, and antibiotic administration. Fluid resuscitation and vasopressor administration should begin immediately, along with intubation, ventilation, and renal replacement therapy, as required [1]. For source control, removal of abscesses, packing, or foreign bodies is recommended. However, a few cases of TSS have been successfully treated without the need to remove foreign bodies [19,20], indicating that TSS can sometimes be managed effectively without surgical intervention. In this case, because the patient did not want to undergo surgery, and her nasal inflammation seemed mild, we attempted to treat her without removing the implant. However, we were uncertain whether the implant was genuinely infected in our case. In terms of antibiotic therapy, a combination of vancomycin and clindamycin may be considered as an initial treatment option, targeting Gram-positive cocci, including *S. aureus *or streptococcal species, which are common causative organisms of TSS. Although no studies have provided guidelines on the duration of antibiotic therapy for TSS [1], antibiotics are typically administered for 14 days.

## Conclusions

This case demonstrates that staphylococcal TSS can occur following rhinoplasty performed for cosmetic purposes. Although TSS is a rare complication of cosmetic rhinoplasty, it is important to recognize the increasing prevalence of plastic surgery worldwide. Early diagnosis and prompt treatment are crucial because TSS is a life-threatening condition. When patients present with a sudden onset of fever, rash, and hypotension, clinicians should consider TSS as a possible diagnosis and inquire about any recent history of cosmetic surgery, despite its rarity as a complication.
